# Postharvest Rot of Pomegranate Fruit in Southern Italy: Characterization of the Main Pathogens

**DOI:** 10.3390/jof8050475

**Published:** 2022-04-30

**Authors:** Annamaria Mincuzzi, Simona Marianna Sanzani, Lluís Palou, Marco Ragni, Antonio Ippolito

**Affiliations:** 1 Department of Soil, Plant, and Food Sciences, University of Bari Aldo Moro, Via Amendola 165/A, 70126 Bari, Italy; simonamarianna.sanzani@uniba.it (S.M.S.); antonio.ippolito@uniba.it (A.I.); 2 Laboratori de Patologia, Centre de Tecnologia Postcollita (CTP), Institut Valencià d’Investigacions Agràries (IVIA), Ctra. CV-315 Km 10.7, 46113 Montcada, Valencia, Spain; palou_llu@gva.es; 3 Department of Agro-Environmental and Territorial Sciences, University of Bari Aldo Moro, Via Amendola 165/A, 70126 Bari, Italy; marco.ragni@uniba.it

**Keywords:** postharvest diseases, *Punica granatum*, *Talaromyces*, *Cytospora*, *Colletotrichum*, *Alternaria*, *Coniella*, *Botrytis*

## Abstract

Pomegranate (*Punica granatum* L.) is an emerging crop in Italy and particularly in southern regions, such as Apulia, Basilicata, and Sicily, due to favorable climatic conditions. The crop is affected by several pathogenic fungi, primarily in the field, but also during postharvest phases. The most important postharvest fungal diseases in pomegranate are gray and blue molds, black heart and black spot, anthracnose, dry rot, and various soft rots. The limited number of fungicides allowed for treatment in the field and the lack of postharvest fungicides make it difficult to control latent, quiescent, and incipient fungal infections. Symptomatic pomegranates from southern Italy were sampled and isolated fungi were morphologically and molecularly characterized. The data obtained revealed that various species of *Penicillium sensu lato* (including *Talaromyces* genus), *Alternaria* spp., *Coniella granati*, and *Botrytis cinerea* were the principal etiological agents of postharvest pomegranate fruit diseases; other relevant pathogens, although less represented, were ascribable to *Aspergillus* sect. *nigri*, *Colletotrichum acutatum sensu stricto*, and *Cytospora punicae*. About two thirds of the isolated pathogens were responsible for latent infections. The results obtained may be useful in planning phytosanitary control strategies from the field to storage, so as to reduce yield losses.

## 1. Introduction

Pomegranate (*Punica granatum* L.) is an ancient shrub that originated in the Transcaucasian-Caspian region [1], and then gradually spread all over the world. Pomegranate fruit, called “balausta”, maintains the residues of the thick floral calyx that are involved in the course of infection, although the leathery and woody rind guarantees physical protection to a certain degree [2]. Pomegranate production worldwide totals about 8.1 million tons, according to the latest data available, and the main producers are India, China, and Iran, which represent 70% of world trade [3]. In Europe, the chief producers are Spain and Italy; while Spanish pomegranate production is focused on export, Italian production is not sufficient to cover national demand [4] and, therefore, Italy imports 4% of the world market [3]. The most exportable cultivars are the Israeli ‘Akko’, the American ‘Wonderful’ and the Spanish ‘Mollar de Elche’, with their related clones, even though royalty-free cultivars promising high-quality products are widespread [5]. Due to their sequential ripening and organoleptic qualities, the former three cultivars are the ones most widely cropped both in Europe and Italy [1,6]. In 2019, total production and total surface area of Italian pomegranate orchards amounted to 14,445.7 tons and 1234 ha, respectively (www.istat.it; accessed on 15 December 2021). About 70% of Italian pomegranate orchards are located in the South, particularly in the Apulia and Sicily regions, which are comparable in both total surface and total production. In Apulia, 374 ha are cultivated, producing 3926 tons of fruit, mainly in Taranto province (Table S1). Favorable weather and the presence of ancient local ecotypes, e.g., “Dente di cavallo” [7], contributed to the success of Apulian pomegranate cultivation; in addition, nutraceutical properties [8], familiarity with the product [6], and a European shipping network favors the exportation of pomegranate as a fresh product [9]. Yield losses of pomegranates might assume great economic relevance. As reported by Murthy et al. [10], in India, a 35% yield loss, i.e., in the field (10%), wholesale (10%), and at retail sites (15%), has been estimated; losses in the field were mainly related to wounds, cracking, and fungal infections, while secondary fungal infections, overripening, dehydration, and other physical damage caused wholesale losses during transport and at retail sites [10]. In a more recent study, also carried out in India, quite similar results on yield losses were obtained [11]. In South Africa, 18% of fruit losses occurred at harvest, mainly due to abiotic damage, while 23% and 21.5% of fruit losses occurred from harvest till the first stage of transportation, and from transportation to marketing, respectively [12,13]. Although many biotic and abiotic factors (temperature, humidity, host variety, etc.) can affect pathogenicity, promising results have been obtained under controlled atmosphere (CA) conditions [14,15]. The main genera of fungal pathogens involved in fruit rot worldwide are *Penicillium*, *Alternaria*, and *Colletotrichum*, causing blue mold, black heart or black spot, and anthracnose, respectively; also, soft rot due to *Coniella granati* and *Botrytis cinerea* is significant [16,17,18,19,20]. Some of these pathogens infect fruit in the field during blossoming (e.g., *B. cinerea* and *Alternaria* spp.), remaining latent until the establishment of optimal growth conditions, these are the so-called “latent pathogens”. Others mainly infect injured pomegranates and are the so-called “wound pathogens” (e.g., *Penicillium* and *Aspergillus* genera) [16]. The risk of secondary infections and the consequent need for prophylactic actions to reduce yield and economic losses seem clear. 

Since there are no overviews of postharvest pomegranate diseases regarding the Italian scenario, the aims of this study were (i) to identify the main postharvest fungal pathogens involved in postharvest loss of pomegranate fruit in southern Italy and (ii) to assess their incidence by evaluating the relevance of wounds and latent pathogens.

## 2. Materials and Methods

### 2.1. Isolate Collection

Between 2015 and 2017, 155 symptomatic pomegranate fruits belonging to cvs. Akko, Mollar de Elche, Wonderful, and Wonderful One, from orchards, local markets, packinghouses, and warehouses located in Apulia and Basilicata regions were analyzed. Rotted fruits were dipped for 2 min in a sodium hypochlorite solution (2%), then rinsed for 1 min in sterile distilled water, and air-dried at room temperature (20 ± 2 °C). Portions of marginal rotted tissues were plated on semi-selective Potato Dextrose Agar (PDA, Conda, Madrid, Spain) amended with 250 mg/L of both streptomycin and ampicillin (Sigma-Aldrich, St. Louis, MA, USA). Plates were incubated in the dark at 24 ± 1 °C for 7 days. Obtained isolates were sub-cultured onto PDA plates achieving pure monoconidial cultures and deposited in the fungal collection of the Department of Soil, Plant, and Food Sciences (DiSSPA), University of Bari Aldo Moro, Italy. 

#### 2.1.1. Morphological Identification

Macro- and micro-morphological features of each single-spore culture grown on PDA were analyzed at genus/species level according to Barnett and Hunter [21]. Furthermore, to highlight distinctive morphological traits, selected isolates were sub-cultured on specific culture media, then incubated under appropriate conditions. Specifically, *Alternaria* spp. isolates were cultured on Potato Carrot Agar (PCA) according to Simmons [22] and *Penicillium* spp. and *Aspergillus* spp. on Malt Extract Agar (MEA, Conda) [23,24]. 

#### 2.1.2. Molecular Identification

To collect mycelium, each isolate was seeded into 20 mL of Potato Dextrose Broth (PDB, Conda), and incubated on a rotary shaker (120 rpm) at 24 ± 1 °C for one week. Aseptically, each one was filtered using a paper-sieved funnel and then dried; the collected mycelium was stored at −20 ± 1 °C until use. Fungal genomic DNA was extracted from 100 mg dried mycelium using a Plant/Fungi DNA Isolation Kit (Norgen, Thorold, ON, Canada) according to the manufacturer’s instructions; DNA purity and quantity were assessed with Nanodrop 2000 (Thermo Fisher Scientific, Waltham, MA, USA). To confirm preliminary morphological identification, specific molecular assays were conducted (Table 1). PCR reactions were run in a T100 Thermal Cycler (Bio-Rad, Hercules, CA, USA), amplicons resolved in 1.7% agarose gel in TBE buffer (1×) stained by GelRed^®^ (Biotium, Landing Parkway Fremont, CA, USA), and then visualized by Gel Doc™ EZ System (BioRad). 

Quantitative real time PCR (qPCR) and High-Resolution Melting (HRM) reactions were run in a CFX96 Touch Real-time PCR Detection System (Bio-Rad). The amount of fluorescence was evaluated at the end of each cycle using CFX-Manager Software v1.6 (Bio-Rad). For HRM assays, the Precision Melt analysis software (Bio-Rad) automatically clustered the amplicons according to their melting profiles. A cut-off genotype confidence percentage value ≥95% was adopted. Putative isolates of *Botrytis* spp. were molecularly identified according to Sanzani et al. [25] with quantitative real-time PCR (qPCR) assay. This, based on a primer/probe system designed on *B. cinerea* InterGenic Spacer (IGS) regions, was arranged for two representative isolates (B1 and B2). A double approach based on β-tubulin portion [26] was used to identify genera and species belonging to *Penicillium sensu lato* (*s.l.*). Soon thereafter, fungal genera were screened by genus-specific PCR primer pair TALF/TALR, which amplified only strains belonging to the *Talaromyces* genus. Furthermore, *Penicillium sensu stricto* (*s.s.*) strains were further discriminated by a specific HRM assay using PPF1/PPR1 primer pair. To distinguish the different species of collected *Aspergillus* sect. *nigri*, a specific HRM assay, described by Mincuzzi et al. [26], was adopted. The specific primer pair HRM-CMDF/HRM-CMDR was designed on a portion of calmodulin gene. *Alternaria* spp. collection was screened applying the specific HRM assay using a specific primer pair designed on the barcoding region OPA1-3 [27]. Isolates belonging to the *Coniella* genus were confirmed by sequencing of the partial ITS region [28]. For the species characterization of *Colletotrichum*, a multi-locus approach [29] was used, based on partial ITS region (ITS5/ITS4), and portions of actin (ACT512F/ACT783R), β-tubulin (Bt2a/Bt2b), glyceraldehyde-3-phosphate dehydrogenase GDF1/GDR1, and glutamine synthetase (GSF1/GSR1) genes. Isolates belonging to other fungal genera (i.e., *Cytospora* spp.) were confirmed by sequencing of the partial ITS region [30].

## 3. Results

### Fungal Pathogens of Pomegranate Fruit

Three-hundred and forty-six fungal colonies were isolated from the examined symptomatic pomegranates. 

**Figure 1:**
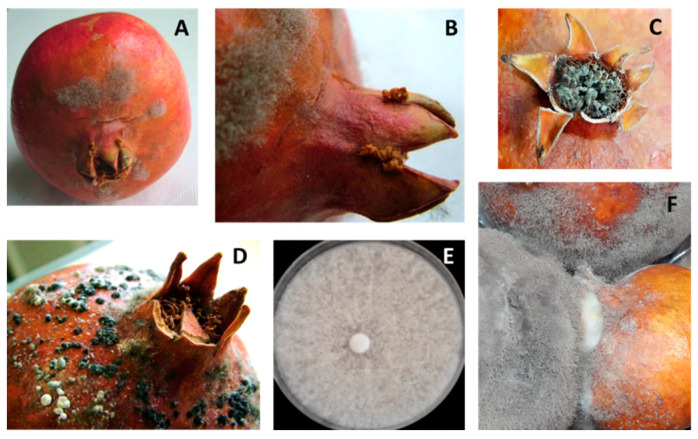
Gray mold caused by *Botrytis cinerea*. (**A**) Early stage of rot. (**B**) Close-up image of sporulated gray mycelia, (**C**) infected stamens and (**D**) black sclerotia. (**E**) *B. cinerea* young colony on PDA. (**F**) Nesting secondary infection between fruits.

*Botrytis* spp. In 21% of the sampled fruit, small tan-colored spots appeared in the crown area, and then rapidly spread over the entire fruit. With time, the spots became darker and softer until rind collapsed, and pomegranates were covered later by a gray fluffy mycelium (Figure 1A–C). Late-stage lesions showed abundant black sclerotia (Figure 1D). In addition, decayed fruit were characterized by softening and browning of the arils, and the growth of a gray inner mycelium. Nesting (Figure 1F) between fruits in close contact was significant. Sixty-four isolates belonging to *Botrytis* spp. were collected. On PDA, both the front and reverse of colonies appeared at first whitish (Figure 1E), then brownish-gray, with the above-mentioned sclerotia arranged in a circle. Conidia were lemon-like in shape, and measured 7.7 ± 2.4 × 6.8 ± 2.5 μm on average; instead, sclerotia of various shapes were 2.9 ± 1.5 × 2.1 ± 0.6 mm. These characteristics matched those of *B. cinerea.* Given the morphological uniformity, two representative isolates, named B1 and B2, were molecularly tested, confirming the morphological identification as being *B. cinerea* (Figure S1). 

**Figure 2:**
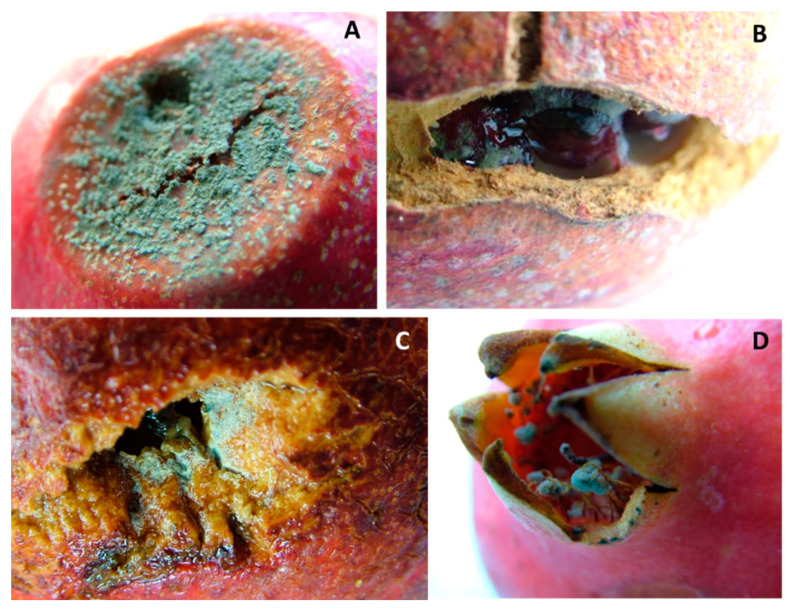
*Penicillium s.l.* decay. (**A**) Necrotic lesion covered with blue-greenish sporification. (**B**) Cracking (**C**) wound and (**D**) infected stamens.

**Figure 3:**
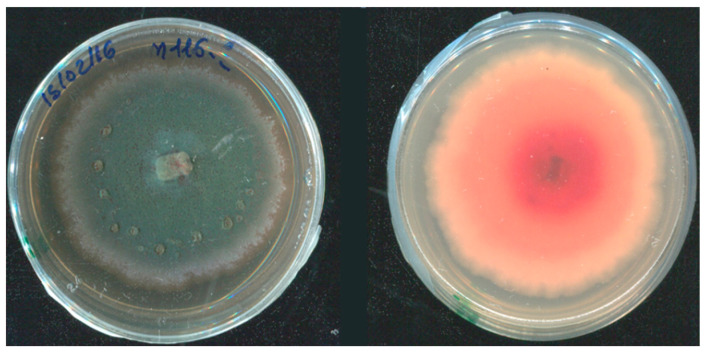
*Talaromyces albobivertillius* on MEA plate: front (**left**) and reverse (**right**); significant is the production of red exudates.

**Figure 4:**
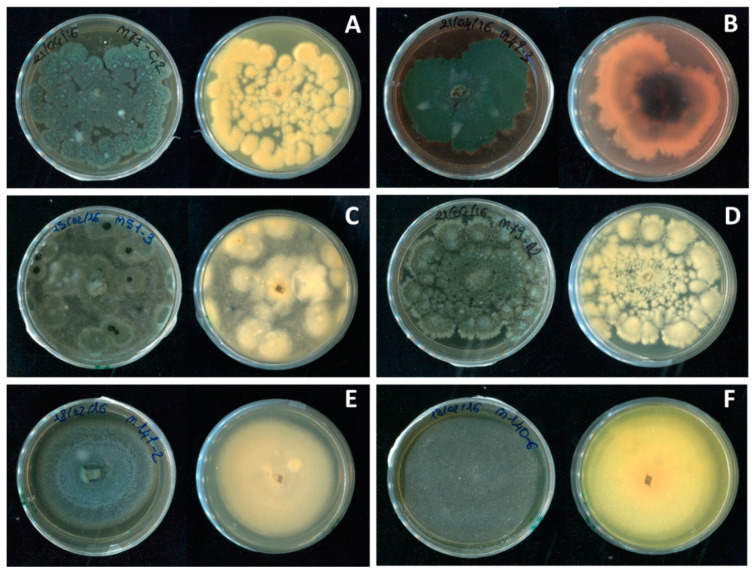
MEA plates of *Penicillium s.s.* species showing macromorphological differences, front (**left**) and reverse (**right**). (**A**) *P. citrinum*, (**B**) *P. pagulum*, (**C**) *P. jhonkrugii*, (**D**) *P. brevicompactum*, (**E**) *P. adametzioides*, and (**F**) *P. glabrum*.

*Penicillium sensu lato* (*s.l.*). In 26% of the collected fruit, brownish circular necrotic lesions of the rind were observed. Over time, these became deeper, darker, and sometimes irregular in shape, and arils softer and browner; finally, masses of blue-green spores grew in/on the infected fruit (Figure 2A). Frequently, stamens and wounds were also covered by this typical blue-green sporification (Figure 2B–D). On MEA, *Penicillium s.l.* colonies showed a texture varying from powdery to velvety or crustose; the surface was blue-green in color, sometimes with concentric whitish margins that differed in thickness and/or wrinkles. Generally, colony reverse was shaded in white-yellow or red-brownish, depending on pigments or secondary metabolite production (Figure 3 and Figure 4B). Even though these features were variable according to species, typical brush-like conidiophores presented spherical and unicellular conidia (2.5–5 µm in diameter) and were visualized as unbranching chains at the tips of the phialides. Often, morphological identification of *Penicillium s.l.* species is difficult, so molecular assays (Table 1) were used in succession. The data obtained showed that 10% of the *Penicillium s.l.* collected from pomegranate fruit was made up of *Talaromyces albobiverticillius* strains (Figure 3), and 90% of *Penicillium sensu stricto* (*s.s.*) species (Figure 4A–F; Table 2). Specifically, six species were recorded within *Penicillium s.s.*: 58% of collected strains were *P. glabrum*, followed by *P. adametzioides* and *P. brevicompactum* that showed an incidence of 25 and 10%, respectively. Other minor species were found: *P. jhonkrugii* (4%), *P. pagulum* (2%), and *P. citrinum* (1%). 

**Figure 5:**
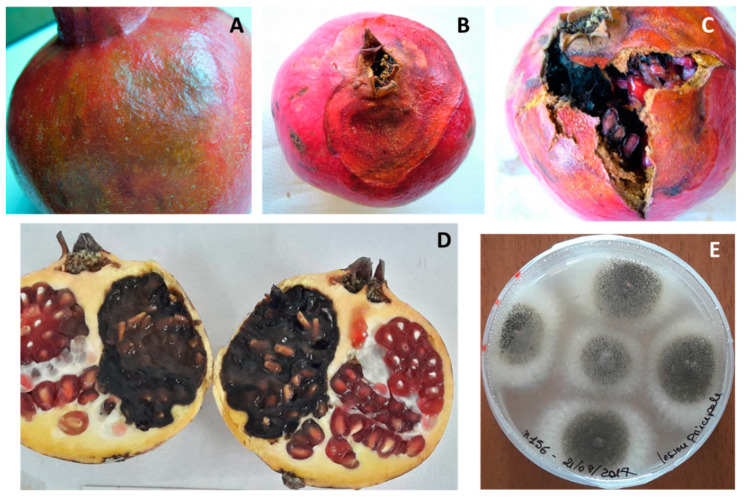
*Aspergillus* sect. *nigri* rot. (**A**) Early and (**B**) advanced stages of the disease. (**C**) Rind cracking of diseased fruit. (**D**) View of internal decay; significant is the soft texture of the tissues. (**E**) Front of a PDA plate of *Aspergillus* spp.

Black aspergilli. In 9% of the collected fruit, internal rot symptoms caused by *Aspergillus* sect. *nigri* were observed. Initially, the rind showed a characteristic concentric discoloration, ranging from yellow to red-brownish shades (Figure 5A,B). Internally, fruit exhibited a soft brownish-black rot in the arils, and sometimes, a powdery black sporulation (Figure 5C,D). On MEA, black aspergilli were cotton-like or velvety in texture (Figure 5E); mycelium was initially white and then appeared black, due to the dark color of sporification. The colony reverse ranged from yellowish to whitish. Hyphae were septate and hyaline. Characteristic conidiophores were aspergillum-shaped and ended in a vesicle with phialides that were attached via the supporting metulae. The conidia, measuring 2–5 µm in diameter, formed radial chains. Since species within black aspergilli clade were very similar in both macro- and micromorphology, molecular confirmation was required for species-level identification (Table 1). According to the applied HRM assay (Table 1), 56% of collected *Aspergillus* spp. were *A. tubingensis*, followed by *A. welwitschiae* that disclosed a 36% incidence. Minor species belonging to the uniseriate group were represented by *A. uvarum* and *A. japonicus*, each showing a 4% incidence (Table 2). 

**Figure 6:**
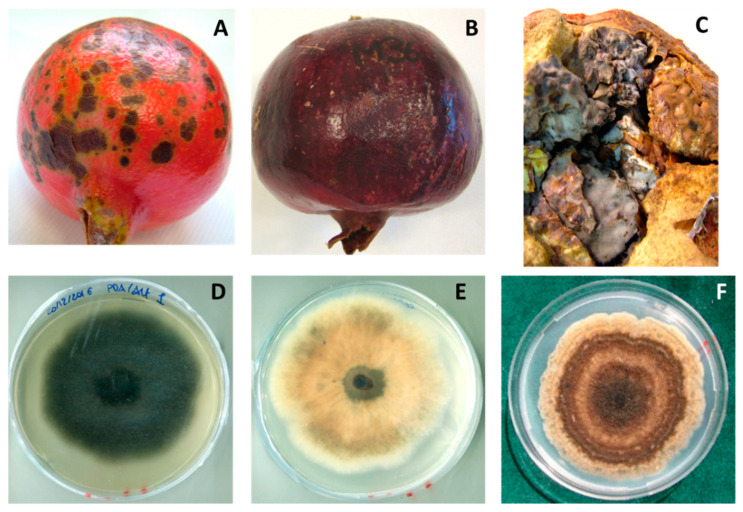
(**A**) Black spot and (**B**) black heart caused by *Alternaria* spp. (**C**) Close-up on heart rot showing mycelium growth. Colonies on PDA of *Alternaria alternata*: (**D**) dark and (**E**) whitish strains. (**F**) Colony of *Alternaria arborescens* on PDA.

*Alternaria* spp. Twenty-four percent of collected pomegranates were infected by *Alternaria* species. Two main diseases were related to this pathogen: black spot (Figure 6A) and black heart (Figure 6B,C). Symptoms of black spot, caused by *Alternaria* spp., consisted of small circular black blotches in pomegranate rind, which corresponded to necrotic areas. Spots were reddish-black in the middle, while the edges were surrounded by yellowish-green halos. This disease was not related to internal rots, so the inner part of the fruit was healthy and edible. Conversely, the rind of black-heart-infected fruit was apparently asymptomatic; sometimes, these fruits showed darker rind, which appeared purplish-deep-red in intensely colored cultivars (Figure 6B). Often, these fruits appeared lighter in weight due to dehydration and aril disintegration caused by rot, and occasionally had an asymmetrical and irregular shape. Internally, the pomegranates exhibited brown and soft arils that turned dry and grayish-black over time (Figure 6C); symptoms of infection spread from the crown-calyx area and later spread over the entire fruit. Colony morphology on PDA was variable (Figure 6D–F); colonies ranged among flat, fluffy, and woolly, and were whitish to brown or black. The dark brown conidia (20 ± 10 μm in diameter) were arranged in branched chains and were oval-ellipsoidal shaped, with 3–5 transverse septa. These traits matched those of the *A. alternata* morphotype *alternata*. Greenish-colored colonies with white margins were observed in other isolates, which had elongated conidia with a long-tapered beak and were characterized by a sporulation pattern, corresponding to the *A. alternata* morphotype *tenuissima*. Pale brown, flat, granulated with undulating margins, and long ellipsoidal conidia, with 1–3 transverse septa, characterized morphotype *limoniasperae* within the *Alternaria alternata* species. These features were further confirmed by the sporulation pattern. The last morphological group displayed colonies ranging from greenish-gray to brown in color and exhibited a lower growth rate. Within this group, the conidia were oval or ellipsoidal with both 1–4 transverse and 1–2 longitudinal septa. These features corresponded to *A. arborescens*.

To confirm the morphological identification of *Alternaria* isolates, an HRM assay was applied (Table 1). Twenty-three isolates were tested, confirming the predominance of the *A. alternata* species, which was distributed among three morphotypes: *alternata* (81%), *tenuissima* (10%) and *limoniasperae* (6%). In addition, the species-complex *A. arborescens* was recorded on 3% of the isolates (Table 2). 

**Figure 7:**
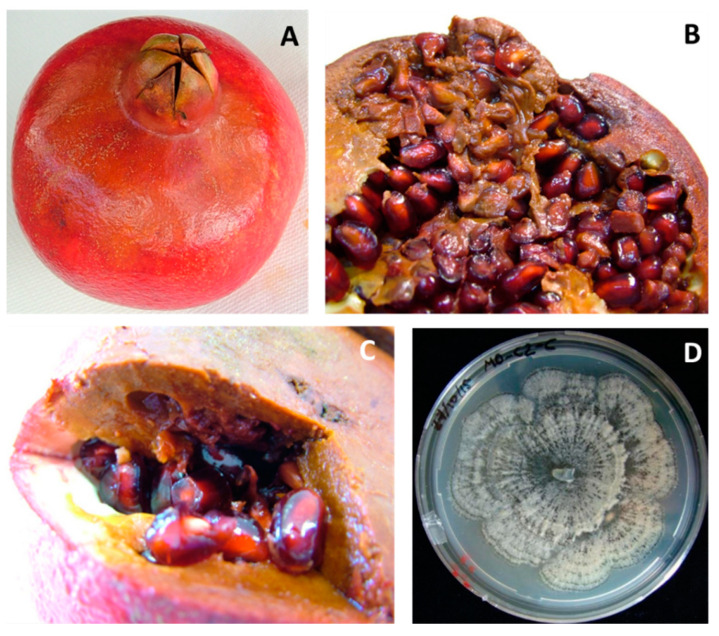
*Coniella granati* rot. (**A**) External symptoms. (**B**) View of internal decay. (**C**) Close-up of the soft rot. (**D**) Colony on PDA plate.

*Coniella* spp. In 15% of the pomegranates, small circular spots appeared on the rind, mainly in the calyx area, which rapidly enlarged and became softer and darker lesions until reaching a brown color (Figure 7A). Mature lesions were covered early by thin whitish mycelia, then by spherical dark brown or black pycnidia. The inner portion of the fruit was decayed, soft, with arils showing softness and browning (Figure 7B,C). Often, damaged areas split. On PDA, colonies were white to creamy, leathery in texture, and covered with abundant dark-greenish-brown to black spherical pycnidia (110 ± 30 μm in diameter, Figure 7D) with thin membranous walls. Hyphae were septate, and conidia hyaline, one-celled, 13.75 ± 3.750 × 3.5 ± 1.5 μm, ellipsoid to fusiform, straight or slightly curved. Given the molecular uniformity, a representative strain was molecularly (Table 1) confirmed as belonging to *C. granati* (Table 2). 

**Figure 8:**
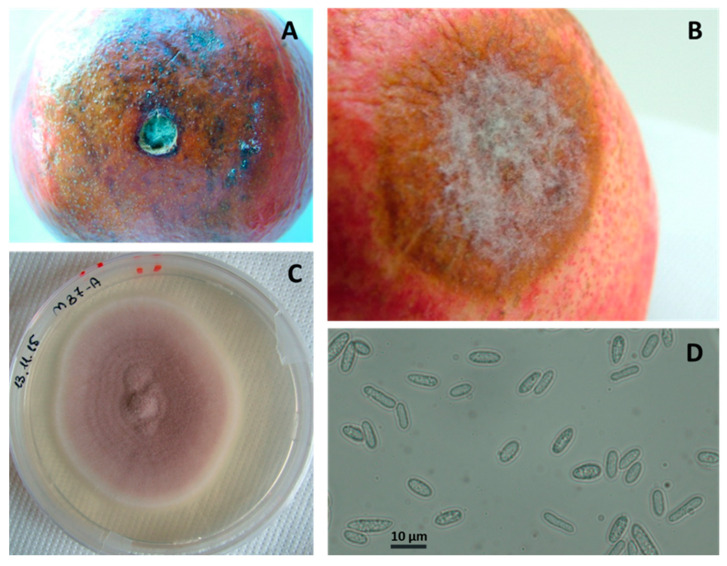
Anthracnose caused by *Colletotrichum acutatum s.s.* Artificial (**A**) and natural (**B**) infections. (**C**) *C. acutatum s.s.* colony grown on PDA plate and (**D**) conidia.

*Colletotrichum* spp. Five percent of fruits presented circular, concentric and brown lesions with darker spots. These soft sunken lesions, typical of anthracnose, merged, increasing in diameter, and then produced white mycelium (Figure 8A,B) and black acervuli. These symptoms were common to several *Colletotrichum* complex species. On PDA, the morphological characteristics of the isolates were similar: fluffy texture, initially whitish with a salmon-grayish reverse and then peachy-pink and covered with pinkish-salmon conidial masses (Figure 8C). Single elliptical-fusiform conidia measured 11.3 ± 2.8 × 4.2 ± 1.1 μm (Figure 8D). The latter feature was important, since conidia measures were generally host specific. To identify the collected isolates at the species level, a molecular multi-locus approach was used (Table 1), and sequences confirmed the representative isolate as being *C. acutatum s.s.* (Table 2).

**Figure 9:**
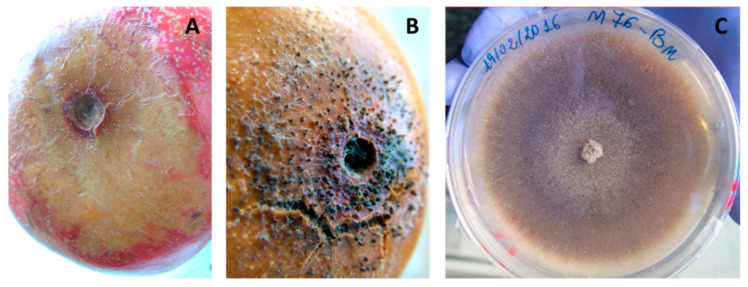
Artificial infections caused by *Cytospora punicae* on pomegranate fruit: (**A**) rot symptom, (**B**) close-up of soft rot showing pycnidia. (**C**) Colony on PDA plate.

*Cytospora* spp. Circular creamy-brownish lesions, centrally darker with soft rind tissue, were observed that corresponded to a subcutaneous area, displaying a yellowish corky appearance (Figure 9A,B). On the PDA plate, monoconidial colonies were at first whitish, then turned olive green and dark brown at maturity (Figure 9C). Globose and dark brown pycnidia (375 ± 125 μm) covered mycelium after 14 days at 24 ± 1 °C; conidia, which appeared allantoid, aseptate, and hyaline, measured 5 ± 1 × 1.5 ± 0.5 μm on average. The molecular assay (Table 1) confirmed *Cytospora punicae* as the representative isolate (Table 2). 

## 4. Discussion

This investigation was organized in three main sections. In the first, a complete description of the most important fungal pathogens of pomegranate fruit from southern Italy was reported. When comparing the number of symptomatic pomegranates (155) with the number of isolates (346), it appeared that several pathogens coexisted in the same fruit. Concerning the incidence of the fungi, the most abundant pathogen of pomegranate fruit was *Penicillium s.l.* (26%), including two different genera, *Talaromyces* and *Penicillium s.s.*; thus, the actual incidence of the latter genus was 24%. The species within *Penicillium s.s.* were *P. glabrum*, *P. adametzioides*, *P. brevicompactum*, *P. jhonkrugii*, *P. citrinum*, and *P. pagulum*. It was *P. glabrum* that showed the major incidence in the genus (59%), confirming numerous previous records; it was reported in Greece [31], Spain [32], Uzbekistan [33], Slovak Republic [34], and Italy [35]. The second most abundant was *P. adametzioides* (24%), already described in Italy [36] and Israel [37]. These two species were pathogenic to pomegranate fruit [31,32,35,36,37], but did not produce mycotoxins; indeed, *P. adametzioides* was proposed as a potential biocontrol agent for Ochratoxin A (OTA) producers, such as *Aspergillus carbonarius*, due to its pigmented secondary metabolites [38]. Within the *Penicillium s.s.* genus, *P. brevicompactum* and *P. johnkrugii* had an incidence of 10 and 3%, respectively; these species were not pathogenic to pomegranates [39], but *P. brevicompactum* produced brevianamide A and mycophenolic acid that could be cytotoxic [40]. The last two collected species belonging to this genus were *P. pagulum* (2%) and *P. citrinum* (1%). *P. pagulum* was recently described by Visagie et al. [41] and, therefore, in previous reports, it may have been named differently or may have been grouped within different species; likewise, *P. citrinum* could have been misidentified with *P. chrysogenum* [42]. For example, species belonging to the *Sclerotiora* section shared several morphological features, such as pigment production [43]; indeed, *P. johnkrugii* showed paraphilias with *P. sclerotiorum* [44] that had already been reported in Spain [16,32]. In addition, according to Houbraken et al. [42,45], *P. citrinum* was synonymized with *P. implicatum*; this possible misidentification might be significant, firstly, because *P. citrinum* produces citrinin [45], a nephrotoxic mycotoxin [46,47], and secondly, because *P. implicatum* was often reported as a pathogen of pomegranate fruit [33,34,48]. A similar misidentification problem could also concern *Talaromyces* genus, which was never reported before on pomegranates [49]. Most of the species belonging to this genus do not produce toxins but red pigments, useful as a potential food coloring [49,50,51], but others, such as *T. islandicus* (syn. *Penicillium islandicus*), produced hepatotoxic and carcinogenic pentapeptides, named cyclochlorotines [52].

*Penicillium s.l.* species are “wound pathogens”; thus, the preferential means for infection were abiotic damage, wounds and lesions caused by harvesting, transport, handling and stocking, fruit cracking, pests, and primary infections caused by other fungi. In addition, being a necrotic dead mass, stamens could represent a secondary inoculum source, even if fruits do not present any physical connection between calyx cavity and inner chambers after fruit setting. Therefore, damaged fruit could be contaminated by colonized stamens [16,34], which are a nutrition source for this necrotrophic genus. For these reasons, during packinghouse operations, their removal is recommended to prevent further infections. 

Comparing the genus abundance within the fungal collection, *Alternaria* spp. was the second most frequent (23% of isolates). Almost all the strains (81%) belonged to *A. alternata* morphotype *alternata*, but some isolates could be ascribed to morphotype *tenuissima* (10%) and *limoniasperae* (6%). The presence of a second species within this genus, *A. arborescens* (3%), which had been already reported in Italy by Aloi et al. [53], with similar incidence, was significant. This occurrence agreed with observations by Kanetis et al. [19] and Luo et al. [54], referring to Greece, Cyprus and California, respectively; these researchers identified *A. alternata* as the main etiological agent of heart rot, as other *Alternaria* species are occasionally responsible for causing the disease. This genus was the causal agent of both black heart and black spot, although each strain could not cause both diseases. Nevertheless, Gat et al. [55] reported that strains of *A. alternata*, isolated from any part of the pomegranate plant and from other hosts, could cause heart rot when inoculated into the fruit. According to Puckett et al. [56] and Ezra et al. [37], the infection occurs in the field during the optimal blooming of hermaphroditic flowers, when petals are open and anthers are not senescent, involving hermaphroditic flowers. Small-spored *Alternaria* conidia are transported into the calyx, then pass through the style and the pollen tube until they reach the mesocarp tissues and the inner part of the fruit. The pathogen remains latent until optimal growth conditions occur, which correspond to mature fruit and high temperature and humidity; then, it spreads towards the upper locules [57]. Finally, knowledge of infection time makes it possible to control the disease more effectively and reduce yield losses; in fact, application of fungicides after the entry and colonization of *Alternaria* conidia is considered useless [56,57]. Ezra et al. [57] supposed that pomegranate black heart etiology was comparable to core rot in apples and that symptomatic and asymptomatic fruits had a different degree of susceptibility that corresponded to physiologically susceptible and resistant plants, respectively. In the same commercial orchard, susceptible and resistant fruits differed in acidity, reflecting the maturity level without any effect due to environment or genotype [57]. On the other hand, pomegranates displayed different susceptibility to *A. alternate*, depending on the cultivar; for example, the Indian cultivars ‘Bhagwa’ and ‘Mridula’ were the most susceptible, while ‘Ruby’ was the least susceptible [58]. Among the 260 secondary metabolites produced by *Alternaria* species, some are mycotoxins hazardous to human and animal health, while others are probably involved in pathogenesis mechanisms eliciting damage in hosts [59]. Some mycotoxins, such as alternariol (AOH), alternariol-monomethyl-ethere (AME), and tenuazoic acid (TA), are found in the juice obtained from pomegranates affected by black spot [60]. 

Soft rots caused by *C. granati* and *B. cinerea* had a comparable incidence of 15% and 21% of isolated fungi, respectively. Despite the similarity of fruit symptoms, showing a soft crown rot that began in the calyx area [17], specific features have to be taken into account. Particularly, *C. granati* is pathogenic for the plant, causing twig blight [61] and collar rot [62,63]; however, only *B. cinerea* could spread by “nesting”. In addition, the former pathogen prefers warmer temperatures (22–32 °C) [64], whereas the latter one could grow even at −2 °C [65], although both need high relative humidity [64]. Furthermore, *C. granati* produces pycnidia, whereas *B. cinerea* produces sclerotia. *C. granati* is widespread in the world [64], especially as an etiological agent of postharvest crown rot [18,32,66]. Despite this, etiological and epidemiological information on this pathogen are scarce. According to Michailides et al. [67] and Thomidis [18], *C. granati* overwinters as pycnidia in mummies and wood littered in orchards; in addition, water sources, such as rainfall and irrigation, could spread pycnidiospora in the field as a source of infection. Being more aggressive, infections could involve the entire fruit more rapidly than *B. cinerea*. According to the morphological and molecular characterization of the most recent studies [68,69], *Coniella* and *Pilidiella*, belonging to Schizoparmeaceae Rossman, fam. nov., should be synonymized. Depending on variety [70], yield losses caused by this pathogen vary as regards severity and, thus, the current activities of researchers are focused on early diagnosis assays, such as Loop-mediated Isothermal Amplification (LAMP) [71].

Concerning *Botrytis*, different species within this genus can cause gray mold. Testempasis et al. [72] reported that *B. cinerea*, *B. pseudocinerea*, and *B. cinerea* group *S* were observed on pomegranates grown in Greece and California. Among these, *B. cinerea* was the most abundant [72], confirming the results from this research. *B. cinerea* is considered the main postharvest fungal pathogen of pomegranate [17,19] due to the large economic losses that it causes [20,73]. Infections occur during blooming, then the pathogen colonizes flower tissues, remaining latent until the presence of free water allows fungal activation and development [72]; furthermore, dead stamens represent a source of secondary inoculum. Other pathways for infection were observed, such as rind wounds, as previously reported by Munhuwehy et al. [20], and nesting, which could favor *Botrytis* colonization. 

In this research, species belonging to *Aspergillus* sect. *nigri* ranked fifth in fungal incidence (9%). Similarly, Kanetis et al. [19] recorded a minor postharvest disease incidence due to black aspergilli. These well-known wound pathogens could also cause internal rot; specifically, infection paths were the same as those disclosed for *B. cinerea* [74]. These fungi were listed worldwide as pomegranate pathogens [20], causing different rates of fruit decay. Most authors detected *A. niger* in previous research [19,20,75], although in this study, different *Aspergillus* species, such as *A. tubingensis*, *A. welwitschiae*, *A. uvarum*, and *A. japonicus*, were identified. The presence of these fungi on pomegranate is relevant from a sanitary point of view, since *A. welwitschiae* strains are potential producers of the mycotoxins fumonisins and ochratoxins [19,76]. Tested isolates proved to be positive for the presence of genes fum8 and fum15 of the fumonisin pathway, but negative for genes involved in OTA biosynthesis [26], although Ferrara et al. [77,78] confirmed the presence of this biosynthetic pathway in *A. welwitschiae*. Some of the strains were tested by HPLC [26] to evaluate the amount of these mycotoxins, showing concentrations of FB over the admitted threshold (CE 1126/07) for other plant products. Regulations still do not report limits for pomegranate and their derivatives.

The existence of wound pathogens suggested the need for good agronomical practices to avoid fruit cracking and strengthen the rind [74]. Caution during harvesting, transport, handling, and packing could reduce further injuries to the fruits. For all the pathogens, optimal storage conditions permitted both the extension of storage time (and consequently greater availability for marketing) and a reduction in economic losses and waste [79,80]. 

Anthracnose symptoms were identified in 5% of the pomegranates, and particularly in 11% of the cold-stored ‘Wonderful’ pomegranates [27]. They were caused by *C. acutatum s.s.*, although most prior reports indicated *C. gloeosporioides* as the causal agent of this disease [81,82,83,84,85]. The detection of species belonging to *C. acutatum* species complex requires a molecular multi-locus approach, and without this, the likelihood of misidentification is high [86]. Therefore, in some instances *C. acutatum s.s.* presence could have been underestimated, which is what happened for *C. tropicale* [87]. When characterizing *Colletotrichum* species causing anthracnose of pomegranate in the southeastern U.S., Xavier et al. [88] demonstrated the presence of six different species, belonging to two species complex: *C. gloeosporioides* and *C. acutatum*; nevertheless, *C. acutatum s.s.* was not recorded. According to Munhuweyi et al. [20], *C. acutatum* could infect pomegranates through conidia produced in infected leaves and disseminated by water and winds. The fungus produced a cutinolytic enzyme that penetrated the healthy rind and, generally, there were no symptoms until ripening; rainfall and damp wind could favor the dissemination of the pathogen when temperatures increase [85]. These conditions, not fully corresponding to Mediterranean weather conditions, can explain the lower incidence of the pathogen in this region [88]. Because of losses caused by this fungus, the high aggressiveness of *C. acutatum*, which was greater on fruits than on leaves, and the different susceptibility of pomegranate cultivars as regards the ripening stage, a mathematical formula to evaluate the virulence was developed [85], and aggressiveness was assessed comparing lesion diameter [88]. The growth and the infectivity of this fungus were favored by optimal environmental conditions, i.e., temperatures ranging between 20 and 30 °C and high humidity [20,82]; in addition, according to Xavier et al. [88], the possibility of cross-infections by different crops cultivated in the same area might exist. 

Finally, a significant minor pathogen was *Cytospora punicae*. This is a well-known causal agent of trunk canker in pomegranate shrubs [20,89,90,91]. On fruits, among *Cytospora* species, *C. annulata* was isolated from harvested pomegranates, but it did not exhibit pathogenicity, as Koch’s postulates were not fulfilled [16]. In contrast, *C. punicae* was recently cited as a causal agent of postharvest fruit rot [30,92].

In addition to pathogenetic fungi (Figure S2), rather well represented was a group of non-pathogenic fungi (Figure S3) that showed a presence of about 10% and included *Bjerkandera adusta*, *Psathyrella candolleana*, and *Purpureocillium lilacinum*, according to morphological and molecular characterization [39]. The first two species were Basydiomycetes; particularly, *B. adusta* was tested in vitro successfully to degrade different industrial dyes at high concentrations [93], and to transform halogenated pesticides using a peroxidase extracted from this white-rot fungus [94]. Biotechnological applications based on *P. lilacinum* were discussed because of its potential pathogenicity in immune-suppressive and immune-compromised individuals [95,96]; therefore, its utilization as a biocontrol agent was uncertain [97,98].

## 5. Conclusions

Based on the obtained results, pomegranate yield losses are mainly caused by latent infections due to pathogens (*Alternaria* spp., *B. cinerea*, and *C. granati*, above all) infecting fruits during the blooming stage. Indeed, during flowering, treatments in the field are of basic importance to reduce the loss of ripe fruit that have reached a very high added value after cultivation, harvesting, storage, and marketing. Considering its general abundance, the most important pathogen was *Penicillium s.l.*, strictly related to abiotic damage, such as wounds and lesions, often caused by bad handling. 

In conclusion, the results obtained during our research suggest that it is vital to identify and disseminate good agricultural practices from the field until the time when the fruits are sold, in order to preserve the quality of pomegranates and extend their postharvest life, i.e., careful harvesting, transport, handling and storing of fruits, and compliance with health and hygiene standards. In addition, this research may represent a benchmark that could be useful in the identification of chief postharvest diseases and related etiological agents of pomegranate fruit. 

## Figures and Tables

**Table 1:** Pomegranate pathogens, primer name, target region, primer sequence, type of assay, and reference for their characterization.

Genus	Primer Name	Gene	Sequence (5′-3′)	Assay	Source
*Botrytis*	BcF	Intergenic spacer	TGTAATTTCAATGTGCAGAATCC	qPCR	[25]
	BcR		TTGAAATGCGATTAATTGTTGC		
	BcP		FAMCCGGTGAGCCCAGGTCACCT		
*Penicillium sensu lato*	Bt2a	β-tubulin	GGTAACCAAATCGGTGCTGCTTTC	PCR	[26]
	Bt2b		ACCCTCAGTGTAGTGACCCTTGGC		
*Penicillium sensu stricto*	PPF1		GAGCGYATGAACGTCTACTT	HRM	
	PPR1		ACVAGGACGGCACGGGGAAC		
*Talaromyces*	TALF		CGAACAATCGAAAAGCAACC	PCR	
	TALR		TACTTTTTTCATGGTCCTTCG		
*Aspergillus*	CMD5	Calmodulin	CCGAGTACAAGGARGCCTTC	PCR	
	CMD6		CCGATRGAGGTCATRACGTGG		
	HRM-CMDF		ATAGGACAAGGATGGCGATG	HRM	
	HRM-CMDR		AGACTCGGAGGGGTTCTGGC		
*Alternaria*	OPA1–3-L	OPA1–3	CAGGCCCTTCCAATCCAT	PCR	[27]
	OPA1–3-R		AGGCCCTTCAAGCTCTCTTC		
	HRMF		GCCCGATATCATCAGGGCAT	HRM	
	HRMR		ACTCCTACATCTCAAATGCCA		
*Coniella*	ITS5	Internal transcribed spacer	GGAAGTAAAAGTCGTAACAAGG	PCR	[28]
	ITS4		TCCTCCGCTTATTGATATGC		
*Colletotrichum*	ITS5	Internal transcribed spacer	GGAAGTAAAAGTCGTAACAAGG	PCR	[29]
	ITS4		TCCTCCGCTTATTGATATGC		
	ACT512F	Actin	ATGTGCAAGGCCGGTTTCGC		
	ACT783R		TACGAGTCCTTCTGGCCCAT		
	Bt2a	β-tubulin	GGTAACCAAATCGGTGCTGCTTTC		
	Bt2b		ACCCTCAGTGTAGTGACCCTTGGC		
	GDF1	Glyceraldehyde-3-phosphate dehydrogenase	GCCGTCAACGACCCCTTCATTGA		
	GDR1		GGGTGGAGTCGTACTTGAGCATGT		
	GSF1	Glutamine synthetase	ATGGCCGAGTACATCTGG		
	GSR1		GAACCGTCGAAGTTCCAC		
*Cytospora*	ITS1	Internal transcribed spacer	TCCGTAGGTGAACCTGCGG	PCR	[30]
	ITS2		GCTGCGTTCTTCATCGATGC		

**Table 2:** Incidence of each recorded fungal species and accession numbers of strain sequences deposited in GenBank. “N.A”, not available.

Genus	Species	Incidence	Accession No.	Source
*Botrytis*	*cinerea*	21%	N.A.	This study
*Penicillium sensu stricto*	*adametzioides*	6%	MK895700; MK895701	[26]
	*brevicompactum*	2%	MK895702; MK895703	
	*citrinum*	<1%	MK895704	
	*glabrum*	13%	MK895705; MK895706	
	*jhonkrugii*	1%	MK895708; MK895709	
	*pagulum*	<1%	MK895707	
*Talaromyces*	*albobiverticillius*	3%	KY563698	
*Aspergillus*	*japonicus*	<1%	MK919488	
	*tubingensis*	5%	MK919489; MK919490	
	*uvarum*	<1%	MK919493	
	*welwitschiae*	3%	MK919491; MK919492	
*Alternaria*	*alternata*	23%	*submitted*	This study
	*arborescens*	1%	*submitted*	
*Coniella*	*granati*	15%	KU821701	[28]
*Colletotrichum*	*acutatum sensu stricto*	5%	MF581923; MF581920; MF581919; MF581921; MF581922	[29]
*Cytospora*	*punicae*	<1%	KY496629	[30]

## Data Availability

Not applicable.

## Supplementary Materials

The following supporting information can be downloaded at: https://www.mdpi.com/article/10.3390/jof8050475/s1, Table S1: Apulian surface area and production of pomegranate orchards; Figure S1: qPCR assay of *B. cinerea* identification; Figure S2: Pathogenicity tests of proved pathogens; Figure S3: Pathogenicity tests of putative pathogens.
