# Supplementary material for: Postharvest Rot of Pomegranate Fruit in Southern Italy: Characterization of the Main Pathogens
Source: J Fungi (Basel). 2022 Apr 30;8(5):475. doi: 10.3390/jof8050475 (PMC9143415; doi:10.3390/jof8050475)
Supplement: Supplementary file 1 [file jof-08-00475-s001.zip › jof-1673770-supplementary.pdf]

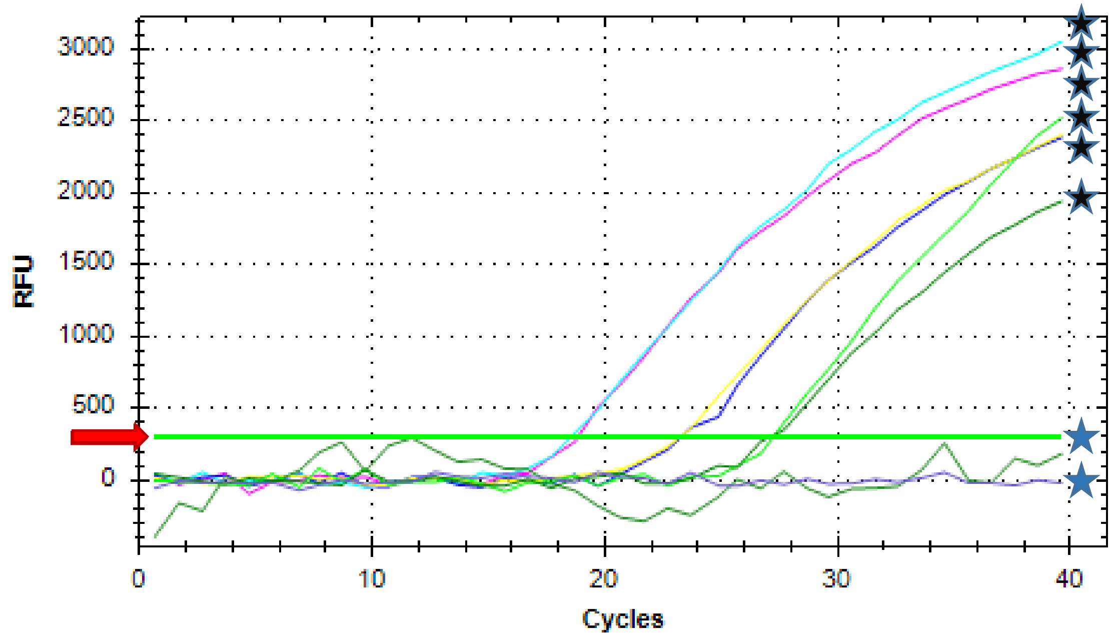

Figure S1. Melting curve profiles generated following real-time amplification in *Botrytis cinerea* assay. Threshold cycle (CT) is indicated by green straight line. *B. cinerea* and positive controls (black star); *Coniella granati,* negative controls (blue star).

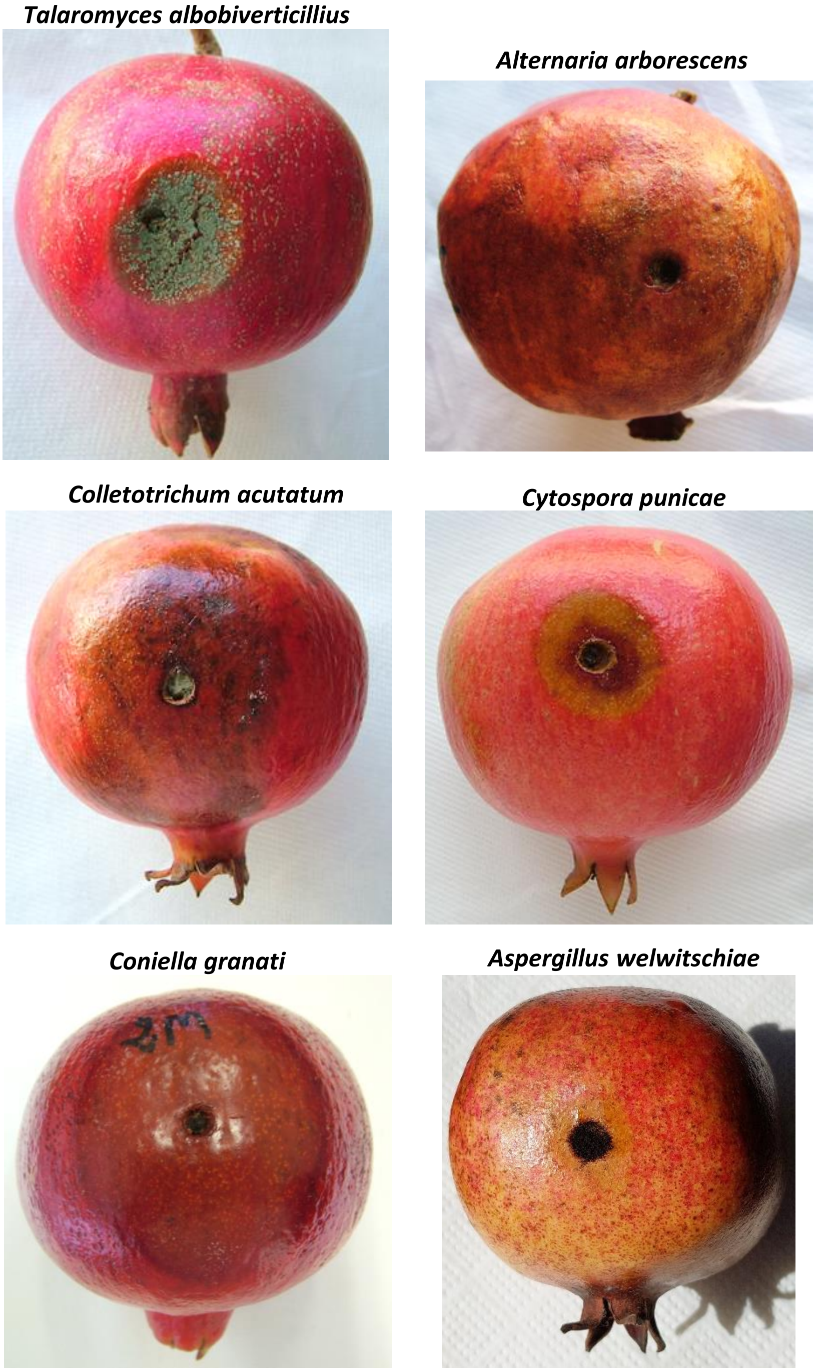

Figure S2. Pathogenicity test on pomegranates inoculated with main fungal pathogens. Species name is reported on the top of related fruit. Fruit were equatorially inoculated by mycelial plugs from actively growing single spore colonies, then incubated at 24±1 °C and 100% RH.

Pathogenicity tests of putative pathogens.

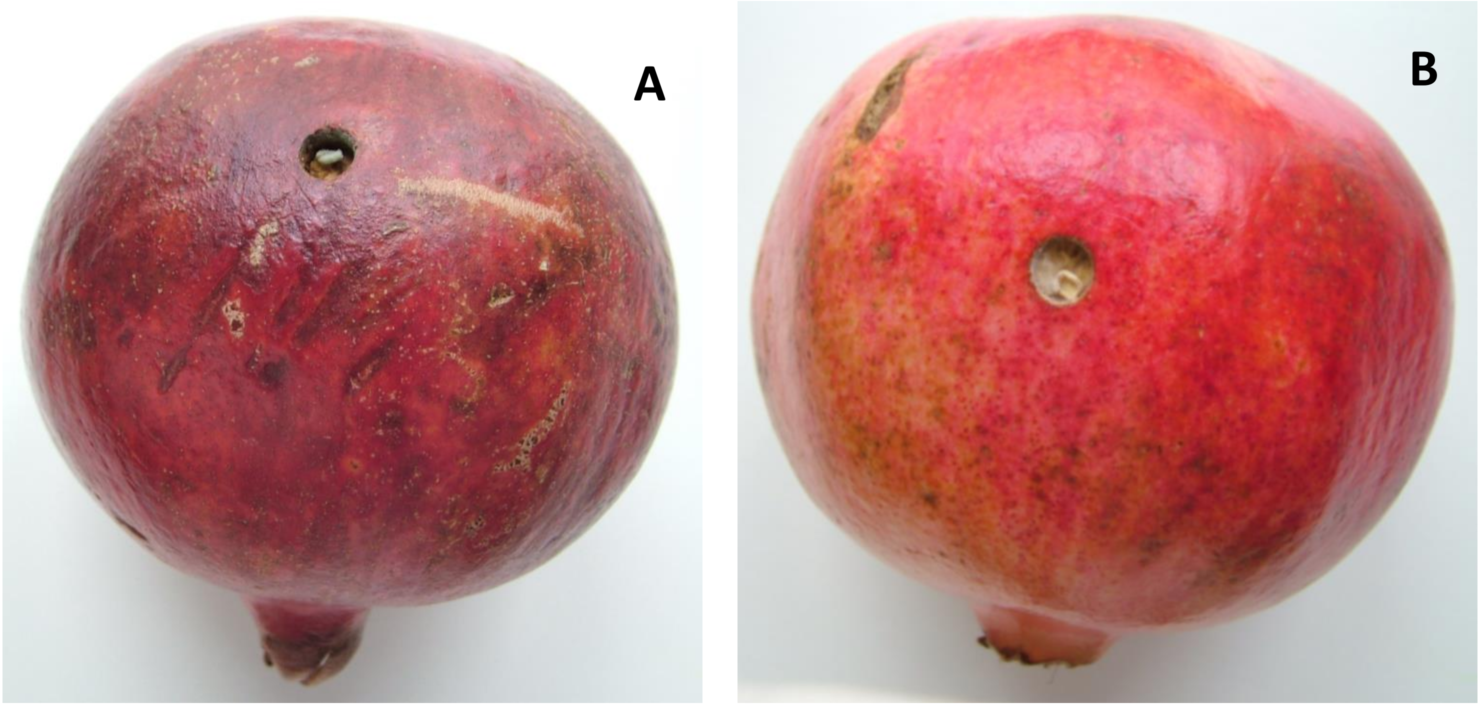

Figure S3. Pathogenicity test on pomegranate inoculated with PDA (control) or mycelial plug. Image displays pathogenicity test arranged for *Bjerkandera adusta*: no infection appear in the control A) and in the inoculated B) fruit, after 14 days of incubation at 24±1 °C and 100% RH. Similar results were obtained for *Psathyrella candolleana* and *Purpureocillium lilacinum*.

Table S1. Pomegranate Apulian surface (ha) and production (ton) in 2018 and 2019. Both parameters are reported as percentage (%). Data refer to Apulia region and its provinces: Foggia (FG), Barletta-Andria-Trani (BAT), Bari (BA), Brindisi (BR), Taranto (TA) and Lecce (LE) (www.istat.it).

	2018				2019			
	Total surface		Total production		Total surface		Total production	
	ha	%	ton	%	ha	%	ton	%
**Apulia**	363	-	3757.7	-	374	-	3925.7	-
**FG**	0	0.00	0.0	0.00	0	0.00	0.0	0.00
**BAT**	1	0.28	0.7	0.02	2	0.53	0.7	0.02
**BA**	50	13.77	800	21.29	50	13.37	900	22.93
**BR**	12	3.31	96	2.55	12	3.21	100	2.55
**TA**	250	68.87	2250	59.88	250	66.84	2250	57.31
**LE**	50	13.77	611	16.26	60	16.04	675	17.19
